# Relationship between single point insulin sensitivity estimator index and nonalcoholic fatty liver disease in Chinese middle-aged and older adults: a cross-sectional study

**DOI:** 10.3389/fnut.2026.1831943

**Published:** 2026-06-15

**Authors:** Duo Yang, Xiaoxian Huang, Renzhe Lin, Sen Li, Zitian Luo, Huankai Zhang, Longsheng Zhang, Jintao Jiang

**Affiliations:** 1 Department of Anesthesiology, Jieyang People’s Hospital, Jieyang, Guangdong, China; 2 Department of Gastroenterology, Jieyang People’s Hospital, Jieyang, Guangdong, China; 3 First Clinical Medical College, Guangdong Medical University, Zhanjiang, Guangdong, China

**Keywords:** cross-sectional study, insulin resistance, middle-aged and older adults, non-alcoholic fatty liver disease, single point insulin sensitivity estimator

## Abstract

**Background:**

Nonalcoholic fatty liver disease (NAFLD) is a major public health issue, especially in China’s middle-aged and elderly population. Insulin resistance (IR) is a key pathogenic factor, yet its gold-standard assessment methods are complex and invasive, limiting clinical use. The Single Point Insulin Sensitivity Estimator (SPISE) index—based on routine measures such as body mass index (BMI), triglycerides (TG), and high-density lipoprotein cholesterol (HDL-C)—offers a simple, insulin-free surrogate marker for IR. However, evidence for its association with NAFLD in China’s middle-aged and elderly population remains limited.

**Methods:**

This cross-sectional study included 1,592 Chinese adults aged 40–79 years. NAFLD was diagnosed by abdominal ultrasonography. The SPISE index was analyzed as both a continuous variable and in quartiles. To assess its association with NAFLD, multivariable logistic regression models were employed, adjusting for a comprehensive set of covariates covering demographics, lifestyle, comorbidities, and metabolic parameters. A restricted cubic spline (RCS) model evaluated the dose–response relationship. Subgroup analyses and receiver operating characteristic (ROC) curve analysis were conducted to test the robustness and diagnostic performance of the association.

**Results:**

The prevalence of NAFLD was 61.12%. A significant inverse association was found between the SPISE index and NAFLD prevalence. In the fully adjusted model, each 1-unit increase in the SPISE index was associated with a 45% lower odds of NAFLD. Participants in the highest SPISE quartile (Q4) had an 86% lower odds of NAFLD compared to those in the lowest quartile (Q1). RCS analysis confirmed a linear inverse dose–response relationship. This inverse association remained consistent across all pre-specified subgroups, including age, gender, BMI, and metabolic status. Furthermore, the SPISE index demonstrated superior diagnostic performance for NAFLD, with an area under the curve (AUC) of 0.78, significantly outperforming its individual components (BMI, TG, or HDL-C alone).

**Conclusion:**

In this Chinese middle-aged and older adult population, a higher SPISE index was independently associated with a significantly lower prevalence of NAFLD, showing a clear dose–response relationship. As a low-cost tool calculated from routine clinical measures, the SPISE index shows promise for the early identification and risk stratification of NAFLD in community and clinical settings.

## Introduction

Nonalcoholic fatty liver disease (NAFLD) has become the most prevalent chronic liver disease globally, posing a significant public health challenge (1). The latest epidemiological data indicate that the pooled global prevalence of NAFLD is estimated to be as high as 32.4% (2), with the overall prevalence significantly higher in males than in females. In China, the disease burden of NAFLD is substantial. Studies have shown that the overall prevalence of NAFLD among adults reached 29.6% over the two decades from 1999 to 2018, and it continues to rise with the epidemics of obesity and diabetes mellitus (DM) (3). NAFLD does not exist in isolation as a hepatic condition; its pathophysiological process is closely associated with Metabolic Syndrome (MetS), with the two conditions interacting bidirectionally. NAFLD significantly increases the risk of adverse outcomes such as atherosclerotic cardiovascular disease, chronic kidney disease, and hepatocellular carcinoma (4). Furthermore, the prevalence of NAFLD exhibits notable age-specific characteristics, with the middle-aged and older adult population bearing the heaviest disease burden (5). The prevalence of NAFLD increases with age. Research indicates that among individuals aged ≥40 years in China, the prevalence can reach 36.9% (6). Individuals in this age group often present with multiple metabolic disorders, making them more susceptible to progressing to nonalcoholic steatohepatitis, hepatic fibrosis, or even cirrhosis, which seriously threatens both life and quality of life. Therefore, an in-depth investigation into the early identification and risk factors for NAFLD within the Chinese middle-aged and older adult population holds important practical significance for developing effective prevention and management strategies (7).

The onset and progression of NAFLD is a complex pathophysiological process in which insulin resistance (IR) is widely recognized as the central driving mechanism (8). IR refers to a state of decreased sensitivity of target tissues to the physiological actions of insulin, leading to compensatory hyperinsulinemia. In the liver, IR promotes fat accumulation through multiple pathways. On one hand, it increases lipolysis in peripheral adipose tissue, resulting in a large influx of free fatty acids (FFAs) into the liver (9). On the other hand, hyperinsulinemia itself activates sterol regulatory element-binding protein-1c (SREBP-1c), thereby enhancing fat synthesis in the liver (10). The abnormal accumulation of lipids, particularly diacylglycerol, within hepatocytes activates protein kinase C epsilon, which subsequently interferes with the tyrosine phosphorylation of insulin receptor substrate (IRS) proteins, impairing hepatic insulin signaling. This establishes a vicious cycle where hepatic fat accumulation and worsening IR reinforce each other (11). Furthermore, IR interacts with processes such as chronic low-grade inflammation, oxidative stress, and endoplasmic reticulum stress, collectively driving disease progression from simple hepatic steatosis to nonalcoholic steatohepatitis, hepatic fibrosis, and even cirrhosis (12). A large-scale Mendelian randomization study further confirmed that IR plays a key mediating role in the association between obesity and NAFLD (13). Accurately assessing IR is crucial for understanding the pathological mechanisms of NAFLD and for risk stratification. Currently, the gold standard method for evaluating insulin sensitivity is the hyperinsulinemic-euglycemic clamp (HEC) technique (14). However, this procedure is complex, time-consuming, costly, and invasive. These limitations severely restrict the widespread application of the HEC in routine clinical practice and large-scale epidemiological research (14). Therefore, developing and validating simple, non-invasive, and reliable surrogate indices for assessing IR is of significant practical importance for conducting NAFLD-related research at the population level.

Given the limited applicability of the gold standard method for assessing IR, the HEC, in large-scale epidemiological studies, various surrogate tools based on fasting blood indices have emerged. Among them, the homeostasis model assessment of IR (HOMA-IR) and the triglyceride-glucose (TyG) index are widely used. However, the former relies on insulin assays with considerable variability and higher costs, while the diagnostic performance of the latter remains controversial across different populations (15). In this context, the Single Point Insulin Sensitivity Estimator (SPISE) index was proposed as a novel tool requiring only three easily obtainable routine metrics: body mass index (BMI), triglycerides (TG), and high-density lipoprotein cholesterol (HDL-C). Its formula integrates two core features of IR, obesity and atherogenic dyslipidemia, theoretically providing a more comprehensive reflection of insulin sensitivity status. Recent studies have confirmed that the SPISE index demonstrates good efficacy in identifying IR and MetS, with diagnostic accuracy superior to some traditional indices (16). Given that IR is the core driver of NAFLD, emerging research suggests a significant association between the SPISE index, calculated from routine metabolic parameters, and the likelihood of NAFLD. This association has been observationally validated in several population-based studies. For instance, a lower SPISE index has been linked to a higher prevalence or incidence of NAFLD among populations with childhood and adolescent obesity, as well as in patients with DM (17–19). However, evidence investigating the association between the SPISE index and NAFLD in general adult populations, particularly the Chinese middle-aged and older adults who bear a high burden of the disease, remains limited. The prevalence of NAFLD is persistently high in this Chinese demographic, and their metabolic profiles, disease characteristics, and progression risks may possess unique features (20). Therefore, validating the utility of the SPISE index in this specific population using large-scale data is of significant importance for developing a cost-effective tool for the early risk stratification of NAFLD.

Based on the background of the published research and the existing gap in this field, this study aims to investigate the association between the baseline SPISE index and the prevalence of NAFLD in a Chinese middle-aged and older adult population, utilizing the Dryad public database. The findings are expected to provide preliminary epidemiological evidence for evaluating the potential value of the SPISE index as a simple screening tool for NAFLD in this population.

## Method

### Data source

The data for this study were sourced from Dryad, a well-known international open science data repository. This platform adheres to open access principles and aggregates publicly available research data resources across multiple disciplines. The dataset utilized in this study is associated with a Chinese cross-sectional study published in *BMJ Open* in 2023, entitled “Association of fat-to-muscle ratio with non-alcoholic fatty liver disease: a single-center retrospective study” (21). Researchers can access the original data via the Digital Object Identifier linked to that publication.^1^ The dataset comprises a study population who underwent routine health examinations at Wuhan Union Hospital between January 2020 and November 2021 and completed a standardized questionnaire concerning gender, age, tobacco use, alcohol use, medical history, and medication use. In accordance with Dryad’s policies, secondary analysis of publicly available data is permitted to explore new scientific questions within an ethical and legal framework. As the original study had obtained ethical approval from the Ethics Committee of Tongji Medical College, Huazhong University of Science and Technology, and this secondary analysis does not involve the recruitment of new participants, neither additional ethical review nor renewed informed consent was required. All procedures in this study strictly adhered to the ethical standards outlined in the Declaration of Helsinki and were fully compliant with national regulations and institutional guidelines.

### Study population

This study constitutes a secondary analysis of a cross-sectional study based on a Chinese mainland population. The original study was initially established by Yan et al. (21), which continuously recruited 1830 participants aged 40–79 years who voluntarily underwent body composition analysis and hepatic ultrasonography during health examinations. Information on gender, age, tobacco use, alcohol use, disease history, and medication history was collected via questionnaires. Exclusion criteria were as follows: excessive alcohol consumption (>210 g/week for men, >140 g/week for women); a known history of liver diseases (e.g., viral, autoimmune, or drug-induced); acute illness, renal insufficiency (estimated glomerular filtration rate <60 mL/min/1.73m^2^), or active cancer (diagnosed or treated within the past 6 months); use of oral or injectable steroids; and missing data for key biochemical indicators or history interview records. After applying these criteria, 1,592 participants constituted the preliminary analytical cohort.

This study aims to investigate the association between the SPISE index and NAFLD in a Chinese middle-aged and older adult population. As data for BMI, HDL-C, and TG—required for calculating the SPISE index—were complete for all participants, no further exclusions were made. Consequently, all 1,592 participants were included in the final analysis. Furthermore, the dataset provided on the Dryad platform was 100% complete for all analyzed covariates (Supplementary Table 1); thus, no missing data imputation methods were required.

### Data collection and variable definitions

Anthropometric measurements were conducted by trained technicians. Body composition (body weight, fat mass, and muscle mass) was assessed using a multi-frequency bioelectrical impedance analyzer (BIA; Tsinghua Tongfan BCA-2A, China). Participants, after an overnight fast and wearing light clothing, stood barefoot for the assessment. The BIA device was regularly calibrated by the manufacturer. The fat-to-muscle ratio (FMR) was calculated by dividing fat mass by muscle mass. Height was measured, and BMI was calculated as weight in kilograms divided by height in meters squared (kg/m^2^). After resting for at least 10 min, seated blood pressure was measured using an electronic sphygmomanometer (Panasonic EW3106, China). Two measurements were taken with a 5 min interval, and the average was used for data analysis.

Fasting venous blood samples (≥8 h) were processed at the hospital’s central laboratory. Platelet (PLT) count was determined using a Beckman-Coulter hematology analyzer. Biochemical parameters, including total cholesterol (TC), TG, HDL-C, low-density lipoprotein cholesterol (LDL-C), alanine aminotransferase (ALT), aspartate aminotransferase (AST), uric acid (UA), and fasting plasma glucose (FPG), were analyzed on a Beckman AU5800 automated system.

DM was defined as a self-reported history of DM or current use of glucose-lowering medications. Hypertension was defined as a self-reported history of hypertension or current use of antihypertensive medications. The diagnosis of fatty liver was determined by routine abdominal B-mode ultrasonography (Philips IU22, Philips Healthcare) performed by trained technicians. Participants identified as having fatty liver without other hepatic comorbidities were classified as having NAFLD.

In this study, the SPISE index served as the exposure variable and was treated as a continuous variable. Prior to calculation, the fasting lipid parameters (TG and HDL-C) were converted from the dataset’s standard international units (mmol/L) to conventional units (mg/dL) by multiplying by 88.57 and 38.67, respectively. The SPISE index was then estimated according to the formula proposed in the literature (22): SPISE index = [600 * HDL-C (mg/dL)^0.185^]/[TG (mg/dL)^0.2^ * BMI (kg/m^2^)^1.338^]. This calculation formula has been validated in the study population of the Chinese Longitudinal Healthy Longevity Survey (23).

### Statistical method

**Figure 1:**
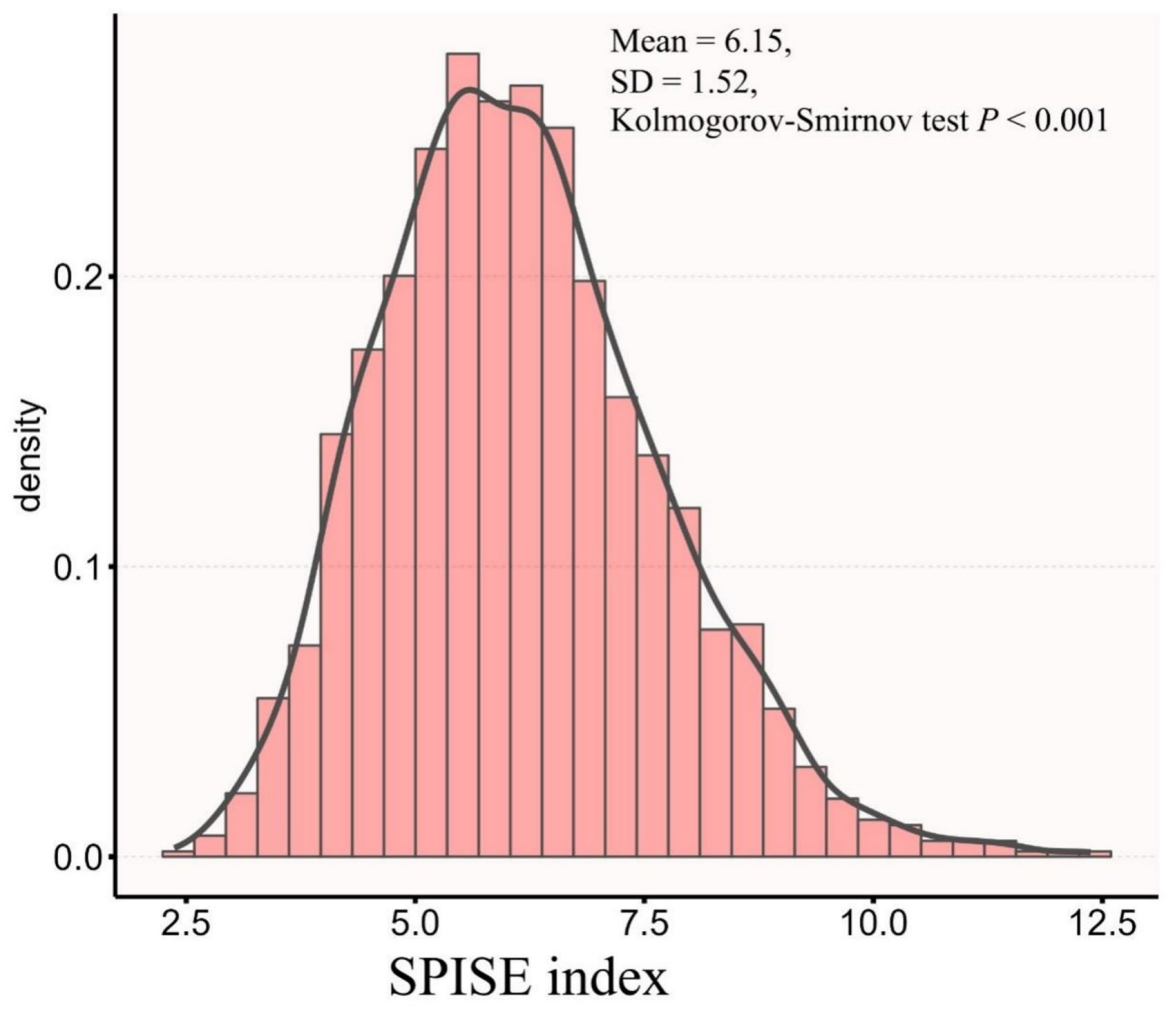
The distribution of the SPISE index.

The distribution of the SPISE index in the study population is shown in Figure 1. Although the Kolmogorov–Smirnov test indicated a statistically significant deviation of the SPISE index from a normal distribution (*p* < 0.001), the large sample size of this study (*n* = 1,592) makes such goodness-of-fit tests highly sensitive to even minor deviations. In practice, given that the data distribution is approximately normal and that parametric statistical methods (e.g., regression analysis) are generally robust with large samples, the SPISE index was treated as approximately normally distributed in the analyses. To analyze the dose–response relationship between SPISE and NAFLD, all participants were divided into quartiles based on SPISE index: Q1 (*n* = 398, 2.369–5.07), Q2 (*n* = 398, 5.077–6.03), Q3 (*n* = 398, 6.03–7.063), and Q4 (*n* = 398, 7.064–12.374). The normality of continuous variables was assessed using the Kolmogorov–Smirnov test. Normally distributed variables are presented as mean ± standard deviation, while non-normally distributed variables are described as median (interquartile range) [*M* (IQR)]. Categorical variables are presented as frequency (percentage) [*n* (%)]. For between-group comparisons, one-way analysis of variance was used for normally distributed continuous variables with homogeneity of variance; otherwise, the Kruskal-Wallis *H* test was applied. Comparisons of categorical variables were performed using the Chi-square test.

Different logistic regression models were constructed to evaluate the association between the SPISE index and NAFLD, quantified with odds ratios (ORs) and 95% confidence intervals (CIs). The crude model was unadjusted. Model I was adjusted for age, gender, tobacco use, alcohol use, hypertension, and DM. Model II was further adjusted for TC, LDL-C, FMR, PLT, AST, ALT, UA, FPG, SBP, and DBP based on model I. The selection of these covariates was based on their established clinical relevance to NAFLD and their potential confounding effects as reported in previous literature. To prevent model instability due to the inclusion of multiple variables, we assessed multicollinearity using the Variance Inflation Factor (VIF). All VIF values were less than 2.5 (Supplementary Table 2), indicating no significant multicollinearity among the adjusted covariates. To test for the linear dose–response trend across the SPISE index quartiles, the median value of each quartile was assigned to the respective participants, and this new variable was treated as a continuous variable in the logistic regression models to calculate the *p*-for trend. An RCS model with four knots located at the 5th, 35th, 65th, and 95th percentiles of the SPISE index was employed to assess the potential non-linear dose–response relationship between the SPISE index and NAFLD. Subgroup analyses were conducted to evaluate potential effect modifications by age, gender, BMI (<28 kg/m^2^ vs. ≥28 kg/m^2^), tobacco use, alcohol use, hypertension, DM, TC (<6.2 mmol/L vs. ≥6.2 mmol/L), TG (<2.3 mmol/L vs. ≥2.3 mmol/L), HDL-C (<1.0 mmol/L vs. ≥1.0 mmol/L), and LDL-C (<4.1 mmol/L vs. ≥4.1 mmol/L). Within each subgroup, logistic regression analyses were uniformly adjusted for the covariates in Model II, except for the stratification variable itself. Interaction tests were performed to evaluate the consistency of the association across these subgroups. The diagnostic performance of the SPISE index and its individual components (TG, HDL-C, and BMI) for NAFLD was evaluated using receiver operating characteristic (ROC) curve analysis. Pairwise comparisons of the AUC between different variables were performed using the DeLong’s non-parametric test. To further comprehensively evaluate the clinical utility of the SPISE index, we also calculated the metabolic score for insulin resistance (METS-IR) and the TyG index for comparison. The diagnostic performance of these indices for NAFLD was compared using ROC curve analysis.

### Statistical software

All results are reported in accordance with the STROBE statement. Statistical analyses were performed in the R environment (version 4.2.2; https://www.R-project.org/) and the Free Statistics Analysis Platform (version 2.1.1; https://www.clinicalscientists.cn/freestatistics). A two-tailed *p*-value of less than 0.05 was considered statistically significant. As the present study constitutes a secondary analysis of pre-existing data, no formal *a priori* sample size calculation was performed.

## Results

### Baseline characteristics of the study population

This study included a total of 1,592 Chinese middle-aged and older participants, comprising 1,148 men (72.11%) and 444 women (27.89%). Participants’ ages ranged from 40–79 years, with the highest number of individuals in the 50–59 age group (709 persons, 44.54%). The overall prevalence of NAFLD was 61.12% (973/1592). Based on the quartiles of the SPISE index, the total population was divided into four groups (Q1–Q4), with 398 individuals in each group. The overall and group-stratified baseline characteristics of the study population are shown in Table 1. As the SPISE index quartile increased (from Q1 to Q4), there were significant differences in the distribution of gender and age among participants (*p* < 0.001). The proportion of women progressively increased from 12.31% in the Q1 group to 51.26% in the Q4 group. Regarding the age distribution, variations were observed across the different groups in each age range, as detailed below: Participants aged 40–49 years showed the highest proportion in the Q1 group (26.13%), the lowest in the Q3 group (16.83%), followed by a rebound in Q4 (24.62%). The proportion of participants aged 50–59 years overall exhibited a decreasing trend with ascending SPISE index quartiles, declining from 48.99% in Q1 to 37.94% in Q4. The proportions of participants aged 60–69 years and 70–79 years were relatively higher in the Q3 and Q4 groups, while dropping to the lowest in Q1 (18.84 and 6.03%, respectively).

**Table 1:** Baseline characteristics of the study population by SPISE index quartiles.

Variables	Total (*n* = 1,592)	SPISE index				
		Q1 (*n* = 398)	Q2 (*n* = 398)	Q3 (*n* = 398)	Q4 (*n* = 398)	*p*-value
SPISE index, mean ± SD	6.15 ± 1.52	4.35 ± 0.54	5.55 ± 0.27	6.52 ± 0.29	8.18 ± 0.95	< 0.001
Age (years), *n* (%)						< 0.001
40–49	354 (22.24)	104 (26.13)	85 (21.36)	67 (16.83)	98 (24.62)	
50–59	709 (44.54)	195 (48.99)	179 (44.97)	184 (46.23)	151 (37.94)	
60–69	360 (22.61)	75 (18.84)	92 (23.12)	95 (23.87)	98 (24.62)	
70–79	169 (10.62)	24 (6.03)	42 (10.55)	52 (13.07)	51 (12.81)	
Gender, *n* (%)						< 0.001
Female	444 (27.89)	49 (12.31)	82 (20.6)	109 (27.39)	204 (51.26)	
Male	1,148 (72.11)	349 (87.69)	316 (79.4)	289 (72.61)	194 (48.74)	
BMI (kg/m^2^), mean ± SD	25.41 ± 2.92	28.52 ± 2.81	25.92 ± 1.79	24.62 ± 1.50	22.58 ± 1.50	< 0.001
FMR, mean ± SD	0.39 ± 0.12	0.42 ± 0.11	0.40 ± 0.11	0.38 ± 0.12	0.37 ± 0.11	< 0.001
UA (μmol/L), mean ± SD	366.75 ± 95.83	409.12 ± 100.63	388.38 ± 88.65	360.01 ± 85.99	309.51 ± 76.30	< 0.001
SBP (mmHg), mean ± SD	130.94 ± 15.82	133.69 ± 14.26	132.82 ± 16.54	130.56 ± 15.73	126.68 ± 15.77	< 0.001
DBP (mmHg), mean ± SD	81.60 ± 11.19	84.35 ± 10.74	82.99 ± 11.16	80.86 ± 10.81	78.20 ± 11.13	< 0.001
FPG (mmol/L), mean ± SD	5.54 ± 1.65	6.16 ± 2.00	5.57 ± 1.54	5.31 ± 1.28	5.13 ± 1.53	< 0.001
TC (mmol/L), mean ± SD	4.48 ± 1.09	4.68 ± 1.10	4.45 ± 0.98	4.35 ± 1.22	4.43 ± 1.04	< 0.001
TG (mmol/L), *M* (IQR)	1.42 (0.98, 2.18)	2.64 (1.97, 4.04)	1.72 (1.31, 2.27)	1.19 (0.94, 1.56)	0.88 (0.69, 1.09)	< 0.001
HDL-C (mmol/L), mean ± SD	1.11 ± 0.32	0.90 ± 0.19	1.02 ± 0.22	1.14 ± 0.27	1.40 ± 0.33	< 0.001
LDL-C (mmol/L), mean ± SD	2.67 ± 0.89	2.60 ± 0.86	2.73 ± 0.86	2.70 ± 0.95	2.64 ± 0.89	0.149
ALT (U/L), *M* (IQR)	21.00 (15.00, 30.00)	28.00 (20.00, 40.00)	23.00 (16.00, 33.00)	20.00 (15.00, 27.75)	16.00 (12.00, 22.00)	< 0.001
AST (U/L), *M* (IQR)	21.00 (17.00, 26.00)	23.00 (18.00, 29.75)	21.00 (17.00, 26.00)	20.00 (17.00, 24.00)	20.00 (17.00, 23.00)	< 0.001
PLT (10^9^/L), mean ± SD	211.15 ± 53.31	209.22 ± 51.73	214.03 ± 54.93	211.11 ± 53.68	210.24 ± 52.91	0.615
Tobacco use, *n* (%)						< 0.001
No	1,054 (66.21)	218 (54.77)	239 (60.05)	279 (70.1)	318 (79.9)	
Yes	538 (33.79)	180 (45.23)	159 (39.95)	119 (29.9)	80 (20.1)	
Alcohol use, *n* (%)						< 0.001
No	1,072 (67.34)	232 (58.29)	230 (57.79)	290 (72.86)	320 (80.4)	
Yes	520 (32.66)	166 (41.71)	168 (42.21)	108 (27.14)	78 (19.6)	
Hypertension, *n* (%)						< 0.001
No	649 (40.77)	107 (26.88)	130 (32.66)	174 (43.72)	238 (59.8)	
Yes	943 (59.23)	291 (73.12)	268 (67.34)	224 (56.28)	160 (40.2)	
DM, *n* (%)						< 0.001
No	1,094 (68.72)	209 (52.51)	265 (66.58)	294 (73.87)	326 (81.91)	
Yes	498 (31.28)	189 (47.49)	133 (33.42)	104 (26.13)	72 (18.09)	
NAFLD, *n* (%)						< 0.001
No	619 (38.88)	46 (11.56)	109 (27.39)	177 (44.47)	287 (72.11)	
Yes	973 (61.12)	352 (88.44)	289 (72.61)	221 (55.53)	111 (27.89)	

Furthermore, compared with the Q1 group, participants in the Q4 group had lower levels of BMI, FMR, UA, SBP, DBP, FPG, TG, ALT, and AST, but higher levels of HDL-C (all *p* < 0.001). TC levels showed a decreasing trend from Q1 to Q3, with a slight increase in Q4, but the differences among groups remained statistically significant (*p* < 0.001). The proportions of current tobacco users, current alcohol users, individuals with hypertension, individuals with DM, and NAFLD patients all showed a progressively lower trend as the SPISE index quartile increased (from Q1 to Q4) (all *p* < 0.001). There were no significant differences in LDL-C and PLT among the SPISE index quartile groups.

According to the baseline characteristics of the population stratified by NAFLD status presented in Table 2, participants in the NAFLD group had a significantly lower SPISE index compared to the non-NAFLD group. Furthermore, the NAFLD group exhibited significantly higher levels of BMI, FMR, UA, SBP, DBP, FPG, TC, TG, ALT, and AST, but a significantly lower level of HDL-C (all *p* < 0.001). The proportions of males, current tobacco users, current alcohol users, individuals with hypertension, and individuals with DM were also significantly higher in the NAFLD group (all *p* < 0.001). However, there were no significant differences between the two groups in age distribution, LDL-C levels, or PLT.

**Table 2:** Baseline characteristics of the study population by NAFLD status.

Variables	Non-NAFLD (*n* = 619)	NAFLD (*n* = 973)	*p*-value
SPISE index, mean ± SD	7.04 ± 1.48	5.58 ± 1.24	< 0.001
Age (years), *n* (%)			0.085
40–49	127 (20.52)	227 (23.33)	
50–59	267 (43.13)	442 (45.43)	
60–69	146 (23.59)	214 (21.99)	
70–79	79 (12.76)	90 (9.25)	
Gender, *n* (%)			< 0.001
Female	229 (37)	215 (22.1)	
Male	390 (63)	758 (77.9)	
BMI (kg/m^2^), mean ± SD	23.99 ± 2.41	26.31 ± 2.86	< 0.001
FMR, mean ± SD	0.38 ± 0.11	0.40 ± 0.12	< 0.001
UA (μmol/L), mean ± SD	337.81 ± 86.51	385.17 ± 96.95	< 0.001
SBP (mmHg), mean ± SD	128.93 ± 15.51	132.21 ± 15.88	< 0.001
DBP (mmHg), mean ± SD	79.73 ± 11.17	82.79 ± 11.05	< 0.001
FPG (mmol/L), mean ± SD	5.19 ± 1.45	5.77 ± 1.73	< 0.001
TC (mmol/L), mean ± SD	4.37 ± 1.01	4.55 ± 1.13	0.001
TG (mmol/L), *M* (IQR)	1.10 (0.82, 1.55)	1.74 (1.17, 2.56)	< 0.001
HDL-C (mmol/L), mean ± SD	1.21 ± 0.36	1.05 ± 0.27	0.001
LDL-C (mmol/L), mean ± SD	2.63 ± 0.86	2.69 ± 0.91	0.193
ALT (U/L), *M* (IQR)	17.00 (13.00, 24.00)	25.00 (17.00, 35.00)	< 0.001
AST (U/L), *M* (IQR)	20.00 (17.00, 24.00)	21.00 (18.00, 27.00)	< 0.001
PLT (10^9^/L), mean ± SD	209.69 ± 53.28	212.08 ± 53.33	0.385
Tobacco use, *n* (%)			< 0.001
No	458 (73.99)	596 (61.25)	
Yes	161 (26.01)	377 (38.75)	
Alcohol use, *n* (%)			< 0.001
No	456 (73.67)	616 (63.31)	
Yes	163 (26.33)	357 (36.69)	
Hypertension, *n* (%)			< 0.001
No	316 (51.05)	333 (34.22)	
Yes	303 (48.95)	640 (65.78)	
DM, *n* (%)			< 0.001
No	493 (79.64)	601 (61.77)	
Yes	126 (20.36)	372 (38.23)	

**Figure 2:**
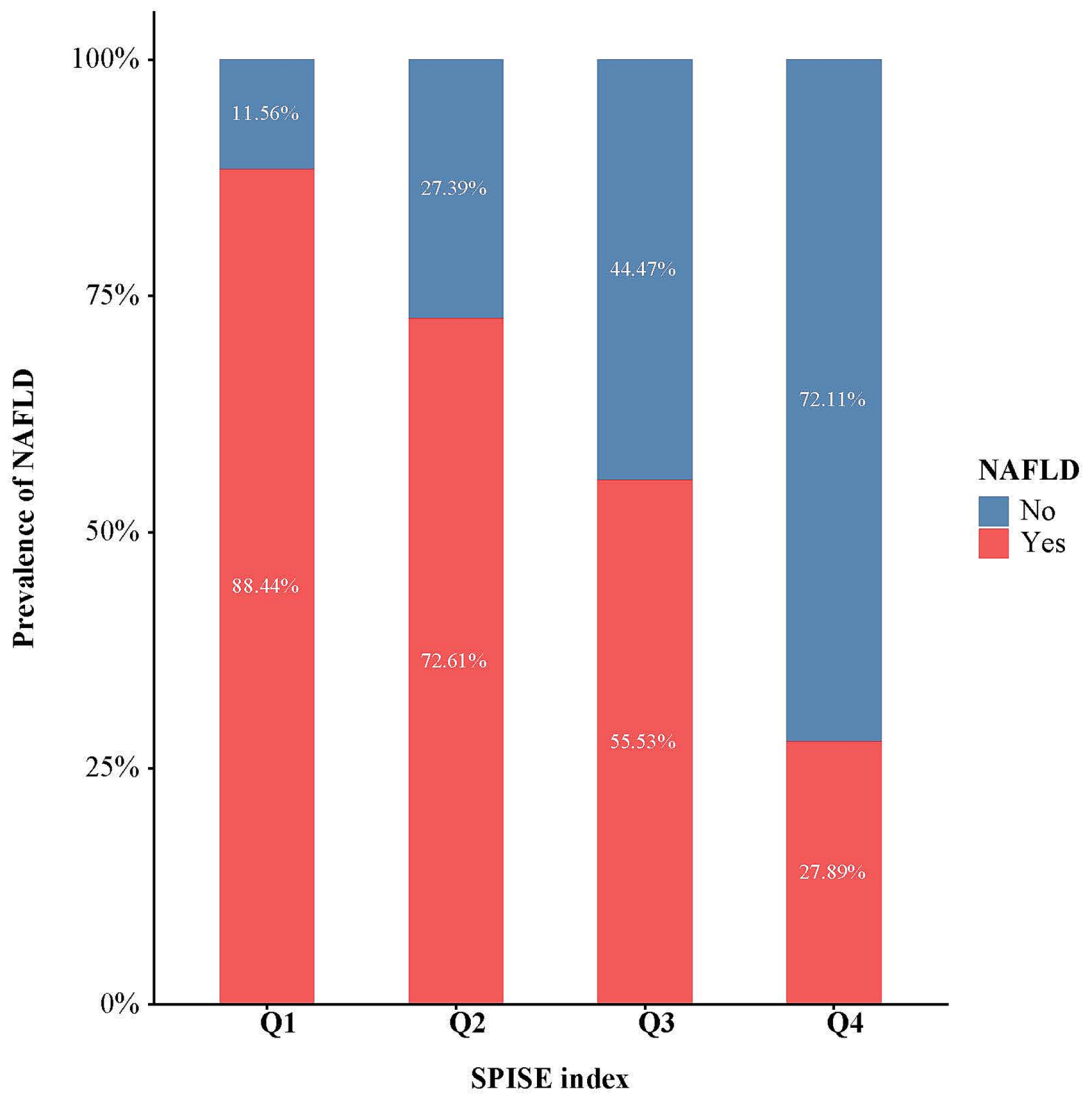
Stacked bar chart of the prevalence of NAFLD. The horizontal axis represents the SPISE index quartiles groups, and the vertical axis represents the prevalence of NAFLD (%). Blue indicates participants without NAFLD, and red indicates participants with NAFLD.

The stacked bar chart in Figure 2 visually illustrates the trend of NAFLD prevalence declining significantly with increasing SPISE index quartiles. Specifically, the prevalence of NAFLD from Q1 to Q4 was 88.44, 72.61, 55.53, and 27.89%, respectively. Compared with participants in the Q1 group, those in the Q4 group exhibited a 60.55% lower prevalence of NAFLD.

### Association between the SPISE index and NAFLD

To assess the association between the SPISE index and the prevalence of NAFLD, we performed multivariable logistic regression analyses (Table 3). In the crude model without adjustment for any covariates, each one-unit increase in the SPISE index was associated with a 55% lower odds of NAFLD (OR = 0.45, 95% CI: 0.41–0.50). Analysis by SPISE index quartiles showed that, using the Q1 group as the reference, the odds of NAFLD were significantly lower in the Q2, Q3, and Q4 groups, with corresponding ORs (95% CI) of 0.35 (0.24–0.51), 0.16 (0.11–0.24), and 0.05 (0.03–0.07), respectively. A significant dose–response trend was observed (*p*-for trend <0.001). This significant inverse association and trend persisted after adjusting for age, gender, tobacco use, alcohol use, hypertension, and DM (Model I). After further adjustment for TC, LDL-C, FMR, PLT, AST, ALT, UA, FPG, SBP, and DBP based on Model I (Model II), the inverse association between the SPISE index and NAFLD prevalence, although attenuated, remained statistically significant (OR = 0.55, 95% CI: 0.49–0.63; *p*-for trend <0.001).

**Table 3:** Association between the SPISE index and prevalent NAFLD, assessed using logistic regression in different models.

Variables	Crude model		Model I		Model II	
	OR (95% CI)	*p*-value	OR (95% CI)	*p*-value	OR (95% CI)	*p*-value
SPISE index (SPISE index quartiles)	0.45 (0.41, 0.5)	< 0.001	0.47 (0.43, 0.52)	< 0.001	0.55 (0.49, 0.63)	< 0.001
Q1	1.00 (Reference)		1.00 (Reference)		1.00 (Reference)	
Q2	0.35 (0.24, 0.51)	< 0.001	0.38 (0.26, 0.56)	< 0.001	0.51 (0.34, 0.77)	0.001
Q3	0.16 (0.11, 0.24)	< 0.001	0.2 (0.14, 0.29)	< 0.001	0.33 (0.22, 0.51)	< 0.001
Q4	0.05 (0.03, 0.07)	< 0.001	0.06 (0.04, 0.1)	< 0.001	0.14 (0.09, 0.23)	< 0.001
*p*-for trend		< 0.001		< 0.001		< 0.001

Crude model: we did not adjust other covariates. Model I: adjusted for age, gender, tobacco use, alcohol use, hypertension, and DM. Model II: further adjusted for TC, LDL-C, FMR, PLT, AST, ALT, UA, FPG, SBP, and DBP based on Model I.

**Figure 3:**
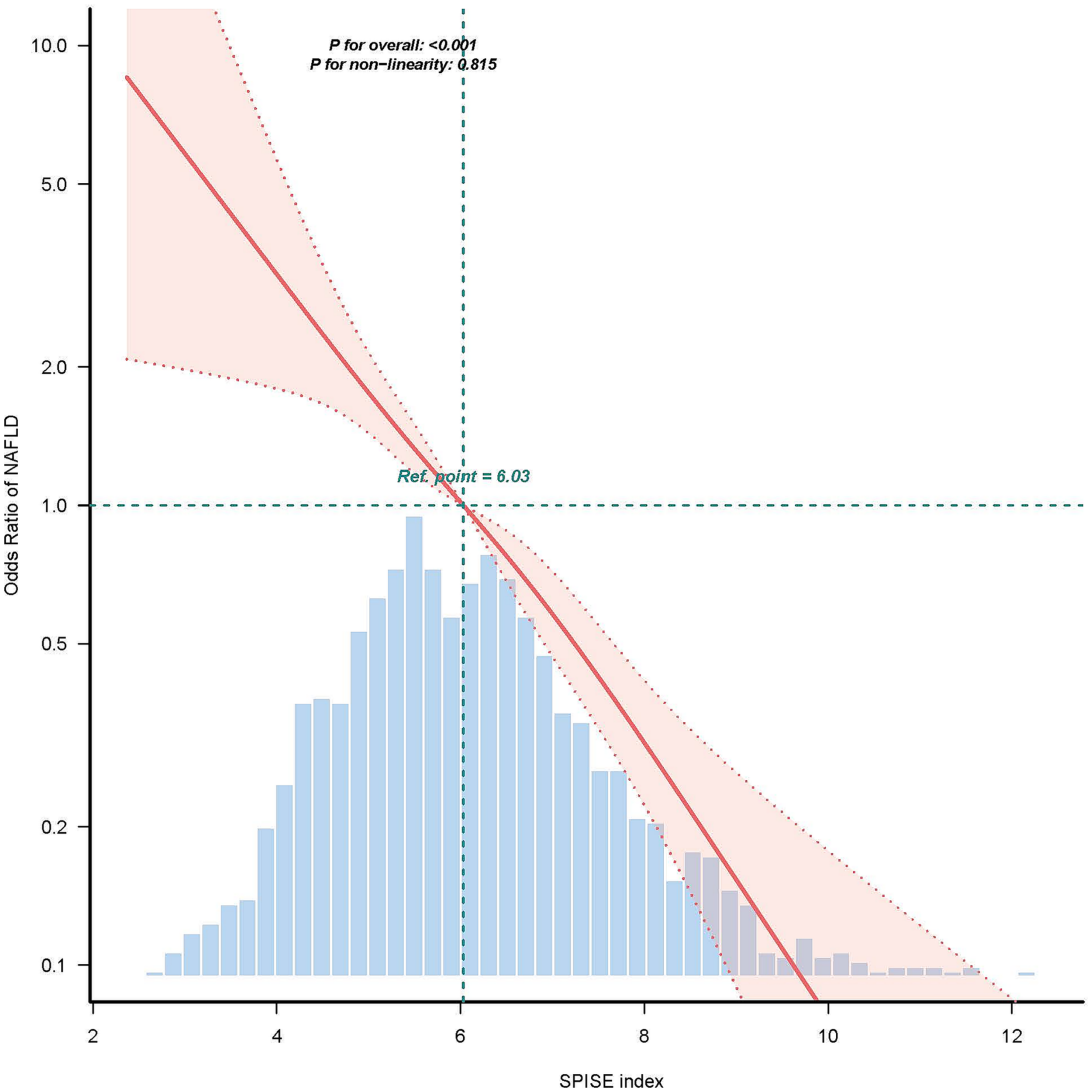
The linear relationship between SPISE index and prevalence of NAFLD. We adjusted age, gender, tobacco use, alcohol use, hypertension, DM, TC, LDL-C, FMR, PLT, AST, ALT, UA, FPG, SBP, and DBP.

Figure 3 presents the RCS curve, which further illustrates a linear inverse association between the SPISE index and the prevalence of NAFLD after adjusting for all covariates in Model II.

### Subgroup analysis

**Figure 4:**
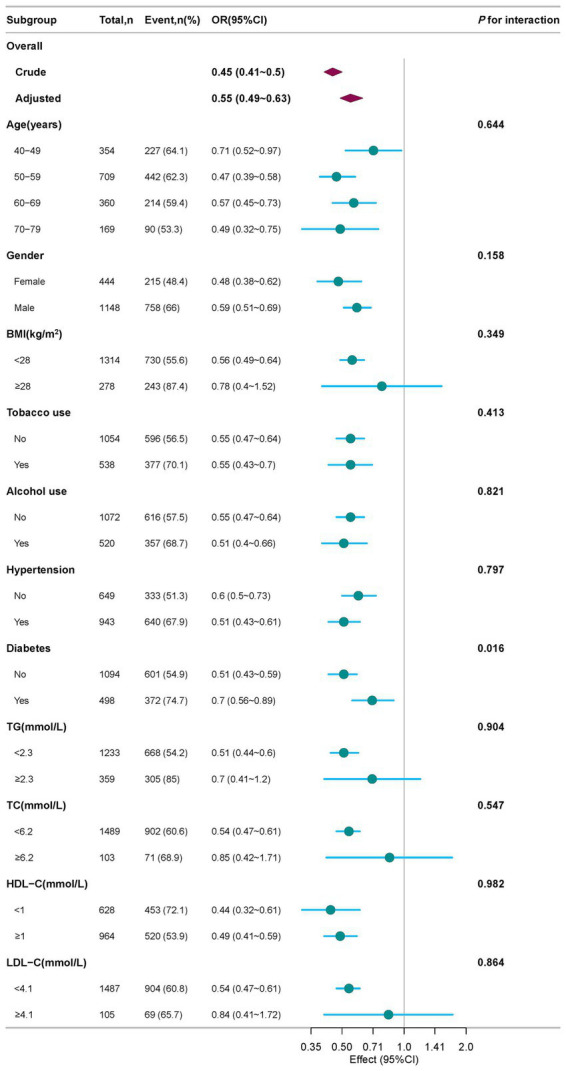
Forest plot of subgroup analysis of the association between SPISE index and prevalence of NAFLD.

According to the forest plot analysis results shown in Figure 4, the adjusted OR point estimates for all pre-specified subgroups were less than 1, indicating a general trend of lower NAFLD likelihood associated with a higher SPISE index. Analyses for each subgroup were adjusted for the same set of covariates as in Model II (excluding the stratification variable itself). However, while the direction of this inverse association was generally consistent with the primary analysis (Table 3), it did not reach statistical significance (i.e., the 95% CI crossed 1.0) in certain strata. Specifically, the association was non-significant among participants with BMI ≥ 28 kg/m^2^, TG ≥ 2.3 mmol/L, TC ≥ 6.2 mmol/L, and LDL-C ≥ 4.1 mmol/L, which may be largely attributable to the relatively small sample sizes within these specific categories.

### Comparative diagnostic performance of the SPISE index and its individual components for NAFLD

**Figure 5:**
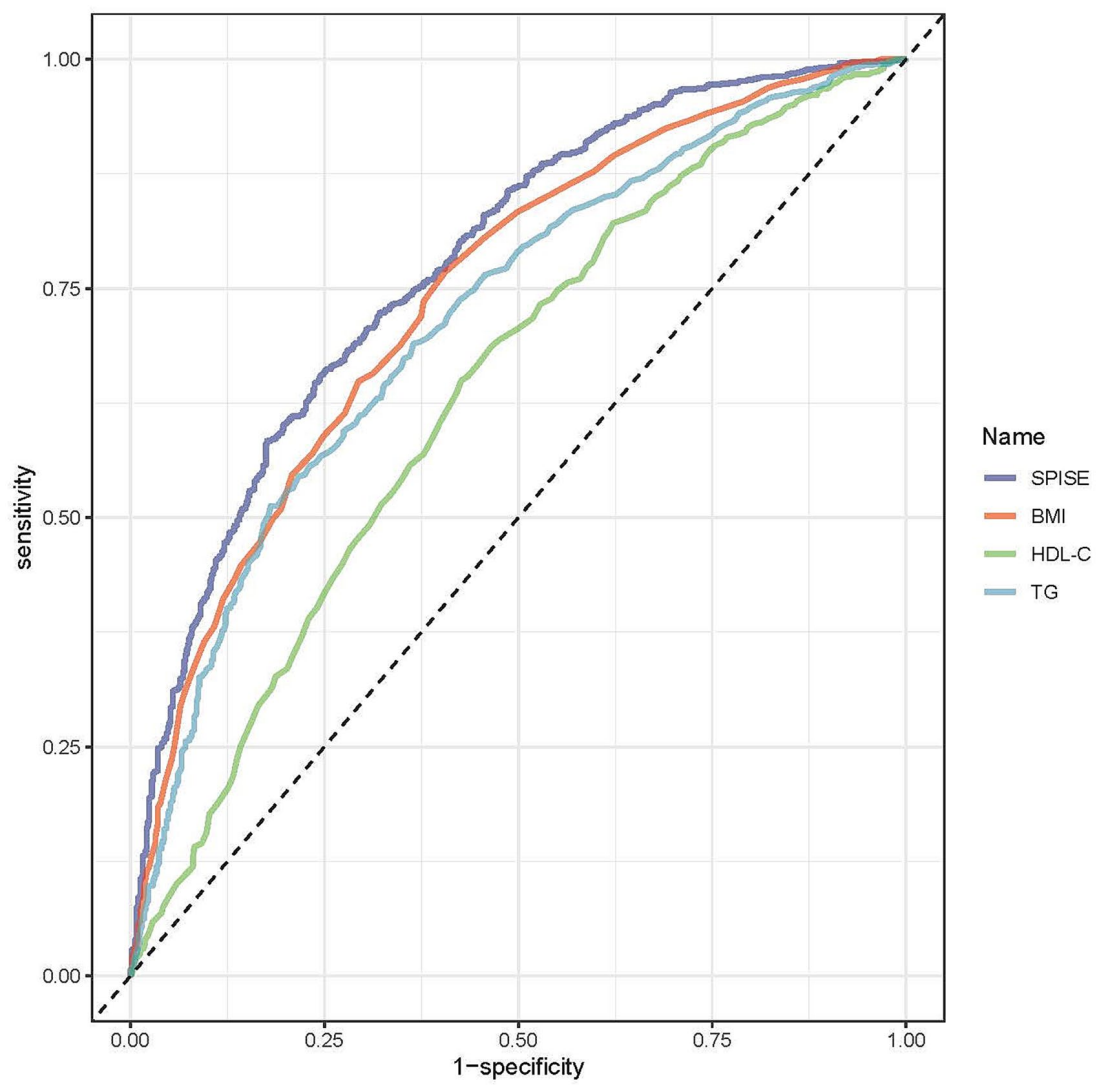
Comparison of the diagnostic performance of SPISE index, BMI, TG, and HDL-C for NAFLD. This figure presents ROC curves evaluating the diagnostic value of the SPISE index and its individual components (BMI, TG, HDL-C) for identifying NAFLD. The results demonstrate that SPISE index (dark blue curve) achieves the highest AUC, indicating that its discriminatory performance significantly outperforms that of BMI (orange-red curve), TG (light blue curve), or HDL-C (green curve) alone. The black dashed line represents the random reference line.

We further compared the diagnostic performance of the SPISE index and its individual components (BMI, TG, and HDL-C) for NAFLD using ROC curve analysis (Figure 5). As shown in Figure 5, Tables 4, 5, the SPISE index demonstrated the best diagnostic efficacy, with an AUC of 0.78 (95% CI: 0.75–0.80). This was superior to BMI alone (AUC = 0.74, 95% CI: 0.72–0.77), TG alone (AUC = 0.72, 95% CI: 0.69–0.74), and HDL-C alone (AUC = 0.64, 95% CI: 0.61–0.67). The optimal cut-off value of the SPISE index for identifying NAFLD was 6.007. At this threshold, the accuracy, sensitivity, and specificity (95% CI) were 0.69 (0.67–0.72), 0.66 (0.63–0.69), and 0.75 (0.72–0.79), respectively. Furthermore, we compared the diagnostic performance of the SPISE index with other established composite insulin resistance indices (METS-IR and TyG index). As shown in Supplementary Tables 3 and 4, the SPISE index demonstrated a statistically superior diagnostic efficacy (AUC = 0.78) compared to both METS-IR (AUC = 0.77, *p* = 0.0044) and the TyG index (AUC = 0.73, *p* < 0.0001).

**Table 4:** Diagnostic performance of individual SPISE index components and the SPISE index for NAFLD identification.

Variables	AUC (95% CI)	Accuracy (95% CI)	Sensitivity (95% CI)	Specificity (95% CI)	Cut off
SPISE index	0.78 (0.75–0.80)	0.69 (0.67–0.72)	0.66 (0.63–0.69)	0.75 (0.72–0.79)	6.007
BMI	0.74 (0.72–0.77)	0.70 (0.68–0.72)	0.59 (0.56–0.63)	0.77 (0.74–0.79)	24.2
TG	0.72 (0.69–0.74)	0.63 (0.61–0.66)	0.82 (0.79–0.85)	0.51 (0.48–0.54)	1.715
HDL-C	0.64 (0.61–0.67)	0.38 (0.36–0.40)	0.43 (0.39–0.47)	0.35 (0.32–0.38)	1.112

This table summarizes the AUC, accuracy, sensitivity, specificity, and optimal cut-off values for the SPISE index and its constituent variables—TG, HDL-C, and BMI—in identifying NAFLD. The composite SPISE index achieved the highest AUC (0.78, 95% CI: 0.75–0.80), followed by BMI (0.74, 95% CI: 0.72–0.77), TG (0.72, 95% CI: 0.69–0.74), and HDL-C (0.64, 95% CI: 0.61–0.67), reinforcing the superior diagnostic utility of the SPISE index demonstrated in Figure 5.

**Table 5:** Pairwise comparison of the AUC between the SPISE index and its individual components for NAFLD identification.

Categories	ΔAUC (95% CI)	SD	*Z*-value	*p*-value
SPISE index *vs*. BMI	0.0335 (0.0160–0.0510)	0.00892	3.756	0.0002
SPISE index *vs*. TG	0.0602 (0.0418–0.0785)	0.00937	6.420	< 0.0001
SPISE index *vs*. HDL-C	0.139 (0.116–0.162)	0.0117	11.866	< 0.0001

## Discussion

Based on cross-sectional data from health examinations of 1,592 individuals in a single-center Chinese middle-aged and older adult population, this study is the first to systematically examine the association between a simple insulin sensitivity estimator index and the prevalence of NAFLD. The analysis revealed that, after extensive adjustment for demographic characteristics, metabolic parameters, and lifestyle factors, a higher SPISE index was independently associated with a significantly lower prevalence of NAFLD, with a clear dose–response relationship. This inverse association showed a generally consistent trend across subgroups stratified by age, gender, BMI, and metabolic status. Although statistical significance was attenuated in a few sub-populations with limited sample sizes, the overall robustness of the relationship between the SPISE index and NAFLD prevalence is evident. Furthermore, the discriminatory performance of the SPISE index for NAFLD was superior to that of any of its individual components, with an AUC reaching 0.78. This indicates that by integrating information on obesity and lipid metabolism, the SPISE index forms a more effective comprehensive risk assessment tool than any single traditional metric. These findings collectively suggest that the SPISE index may serve as a simple and practical metabolic marker for identifying individuals at high likelihood for NAFLD in the middle-aged and older adult populations.

The robust negative association identified here between the SPISE index and NAFLD prevalence is grounded in the well-established role of IR as a central pathophysiological driver of hepatic steatosis (8). An elevated SPISE index—reflecting superior insulin sensitivity—signifies a state of metabolic health where several detrimental pathways are suppressed. Specifically, improved insulin sensitivity reduces the influx of FFAs from adipose tissue lipolysis into the liver (9) and prevents the hyperinsulinemia-driven activation of SREBP-1c, which otherwise fuels hepatic lipid synthesis (10). Furthermore, higher insulin sensitivity is typically associated with mitigated oxidative stress and chronic low-grade inflammation (11, 12), collectively reducing the likelihood of intracellular triglyceride accumulation within hepatocytes (18). Thus, the SPISE index effectively integrates these complex dimensions of lipid and glucose metabolism into a single, clinically accessible metric.

The robust negative association between the SPISE index and NAFLD observed in our study aligns with existing evidence highlighting the index’s utility as a reliable surrogate for systemic insulin sensitivity. Previous research has established that a lower SPISE index is widely associated with various IR-related metabolic and systemic disorders, including periodontitis (24) and cardiovascular diseases in both general and diabetic cohorts (23, 25). More specifically regarding hepatic steatosis, a recent study of Chinese patients with DM also identified the SPISE index as an independent associated factor for NAFLD (18). Our study extends this association to a broader, community-based population regardless of DM status, demonstrating a persistent independent negative association (OR = 0.55). Notably, the diagnostic performance of the SPISE index in our general population (AUC = 0.78) was substantially higher than that previously reported in the diabetic cohort (AUC of 0.268 in men and 0.209 in women) (18). This contrast profoundly suggests that the diagnostic efficacy of such metabolic biomarkers may be significantly influenced by underlying population characteristics. By addressing this gap, our findings provide crucial epidemiological evidence for the utility of the SPISE index as a robust risk stratification tool specifically tailored for the high-risk, general middle-aged and older demographic.

Clinically, the SPISE index presents significant operational advantages as a non-invasive, cost-effective screening tool. By utilizing routine health examination parameters (BMI, TG, HDL-C), it bypasses the need for complex and expensive fasting insulin assessments (such as the HEC technique or HOMA-IR), making it highly accessible for large-scale primary care screenings to rapidly identify individuals at high risk for NAFLD (26). Furthermore, the population-specific quartile thresholds derived from our cohort provide a quantitative basis for precise risk stratification. This composite-index approach is more accurate than relying on single parameters, aiding in the optimal allocation of public health resources toward individuals requiring intensive monitoring (27). Beyond initial screening, the SPISE index holds promise as a dynamic, objective surrogate endpoint for evaluating the efficacy of lifestyle or pharmacological interventions. Tracking changes in the index over time allows clinicians to conveniently monitor improvements in insulin sensitivity, making it a valuable metric for long-term NAFLD management (28). Collectively, its accessibility, pathophysiological relevance, and robust diagnostic performance position the SPISE index as an integrated clinical tool bridging early identification, risk stratification, and longitudinal management in community-based middle-aged and older populations.

This study possesses several notable methodological and demographic strengths. Primarily, it provides direct epidemiological evidence for a high-risk, under-researched demographic by systematically evaluating the association between the SPISE index and NAFLD in Chinese middle-aged and older adults. Methodologically, the integration of sequentially adjusted multivariable models and RCS analysis extends beyond simple binary association testing to establish a robust, approximately linear dose–response relationship, which remained consistent across diverse pre-specified subgroups. Finally, direct ROC comparisons explicitly confirm that the composite SPISE index (AUC = 0.78) yields significantly superior discriminatory power compared to any of its individual metabolic components (BMI, TG, or HDL-C), robustly justifying its added diagnostic value.

Several limitations warrant careful consideration. First, the cross-sectional design precludes establishing temporal sequences or inferring causality. Although biologically plausible, reverse causality—where pre-existing NAFLD induces secondary metabolic disturbances—cannot be ruled out, necessitating validation in prospective cohorts. Second, NAFLD diagnosis relied on abdominal ultrasonography rather than the gold-standard liver biopsy. While cost-effective for large-scale screening, its limited sensitivity for mild steatosis might lead to underestimations of both NAFLD prevalence and the association strength. Third, relying on a single-center, geographically restricted health examination cohort may limit the generalizability of our findings, highlighting the need for multi-center validation. Fourth, despite extensive multivariable adjustments, potential residual confounding from unmeasured factors—such as dietary patterns, physical activity, genetic predispositions, and environmental exposures—remains. Finally, the inherent lack of waist circumference and fasting insulin data in the original dataset prevented comparisons between the SPISE index and other established indices (e.g., LAP, VAI, TWI, or HOMA-IR). However, our primary objective was to establish the epidemiological dose–response association of the SPISE index in this specific demographic, rather than identifying the absolute best screening tool. The comparative superiority observed here requires further validation across diverse cohorts. Finally, we acknowledge the recent international consensus recommending the transition from the term NAFLD to metabolic dysfunction-associated steatotic liver disease (MASLD). While the present study adhered to the established NAFLD diagnostic criteria, future research is warranted to validate the diagnostic performance and clinical utility of the SPISE index within the framework of the updated MASLD definition.

Moving forward, several research directions are warranted to address these limitations. First, prospective cohort studies are needed to establish causality and validate the predictive value of the SPISE index for incident NAFLD. Second, multi-center external validation across diverse populations, ideally utilizing quantitative imaging, is necessary to enhance generalizability and refine optimal diagnostic cut-offs. Third, head-to-head comparisons with established models (e.g., TyG and fatty liver indices) and the integration of the SPISE index with other clinical parameters (e.g., waist circumference and liver enzymes) could help develop optimized, population-specific screening tools. Furthermore, implementation studies should assess the cost-effectiveness of the SPISE index in community screening and its potential as a dynamic biomarker for monitoring interventions. Finally, multi-omics approaches (e.g., metabolomics and genomics) could elucidate the precise molecular mechanisms linking the SPISE index to hepatic fat accumulation.

## Conclusion

This cross-sectional study based on data from a Chinese middle-aged and older adult population demonstrates an independent, significant, and dose–response negative association between the SPISE index and the prevalence of NAFLD. This inverse trend was generally consistent across subgroups with different demographic and metabolic characteristics. Furthermore, the discriminatory performance of the SPISE index for NAFLD was superior to that of its individual component metrics. As an easily obtainable and low-cost surrogate marker of insulin sensitivity, the SPISE index shows good potential for application in identifying individuals at high likelihood for NAFLD within the middle-aged and older adult population.

## Data Availability

The data presented in this study are available in the Dryad repository, doi: https://doi.org/10.5061/dryad.7d7wm3809.

## Footnotes

1
https://doi.org/10.5061/dryad.7d7wm3809

## Glossary

NAFLD: nonalcoholic fatty liver disease
DM: diabetes mellitus
MetS: metabolic syndrome
IR: insulin resistance
FFAs: free fatty acids
SREBP-1c: sterol regulatory element-binding protein-1c
HEC: hyperinsulinemic-euglycemic clamp
HOMA-IR: homeostasis model assessment of IR
TyG: triglyceride-glucose index
SPISE: single point insulin sensitivity estimator
BMI: body mass index
TG: triglyceride
HDL-C: high-density lipoprotein cholesterol
BIA: bioelectrical impedance analyzer
FMR: fat-to-muscle ratio
PLT: platelet
TC: total cholesterol
LDL-C: low-density lipoprotein cholesterol
ALT: alanine aminotransferase
AST: aspartate aminotransferase
UA: uric acid
FPG: fasting plasma glucose
ORs: odds ratios
CIs: confidence intervals
RCS: restricted cubic spline
ROC: receiver operating characteristic
AUC: area under the curve
SD: standard deviation
*M*: median
IQR: interquartile range
SBP: systolic blood pressure
DBP: diastolic blood pressure
METS-IR: metabolic score for insulin resistance
