# Supplementary material for: Relationship between single point insulin sensitivity estimator index and nonalcoholic fatty liver disease in Chinese middle-aged and older adults: a cross-sectional study
Source: Front Nutr. 2026 Jun 15;13:1831943. doi: 10.3389/fnut.2026.1831943 (PMC13310994; doi:10.3389/fnut.2026.1831943)
Supplement: Supplementary file 1 [file supplementary_file_1.docx]

**Supplementary Table 1. Description of missing data.**

| **Variables** | **Non-missing** | **Missing** | **Miss percentage(%)** |
| --- | --- | --- | --- |
| SPISE index | 1592 | 0 | 0 |
| Age | 1592 | 0 | 0 |
| Gender | 1592 | 0 | 0 |
| BMI | 1592 | 0 | 0 |
| FMR | 1592 | 0 | 0 |
| UA | 1592 | 0 | 0 |
| SBP | 1592 | 0 | 0 |
| DBP | 1592 | 0 | 0 |
| FPG | 1592 | 0 | 0 |
| TC | 1592 | 0 | 0 |
| TG | 1592 | 0 | 0 |
| HDL-C | 1592 | 0 | 0 |
| LDL-C | 1592 | 0 | 0 |
| ALT | 1592 | 0 | 0 |
| AST | 1592 | 0 | 0 |
| PLT | 1592 | 0 | 0 |
| Tobacco use | 1592 | 0 | 0 |
| Alcohol use | 1592 | 0 | 0 |
| Hypertension | 1592 | 0 | 0 |
| Diabetes | 1592 | 0 | 0 |
| NAFLD | 1592 | 0 | 0 |

**Supplementary Table 2. Variance inflation factors of covariates included in the multivariable-adjusted model (Model II).**

| **Variables** | **VIF** |
| --- | --- |
| Gender | 1.945 |
| Age | 1.086 |
| Tobacco use | 1.241 |
| Alcohol use | 1.236 |
| Hypertension | 1.186 |
| Diabetes | 1.191 |
| TC | 2.407 |
| LDL-C | 2.363 |
| FMR | 1.854 |
| PLT | 1.061 |
| ALT | 1.392 |
| AST | 1.36 |
| UA | 1.171 |
| FBG | 1.162 |
| SBP | 1.325 |
| DBP | 1.282 |

**Supplementary Figure 1. Comparison of the diagnostic performance of the SPISE index and two other insulin resistance indices (METS-IR and TyG index) for NAFLD.​** The results demonstrate that the SPISE index (blue curve) exhibits the highest discriminatory performance, with its AUC significantly outperforming that of METS-IR (orange curve) and the TyG index (green curve). The black dashed line represents the reference line for a random classifier.

**
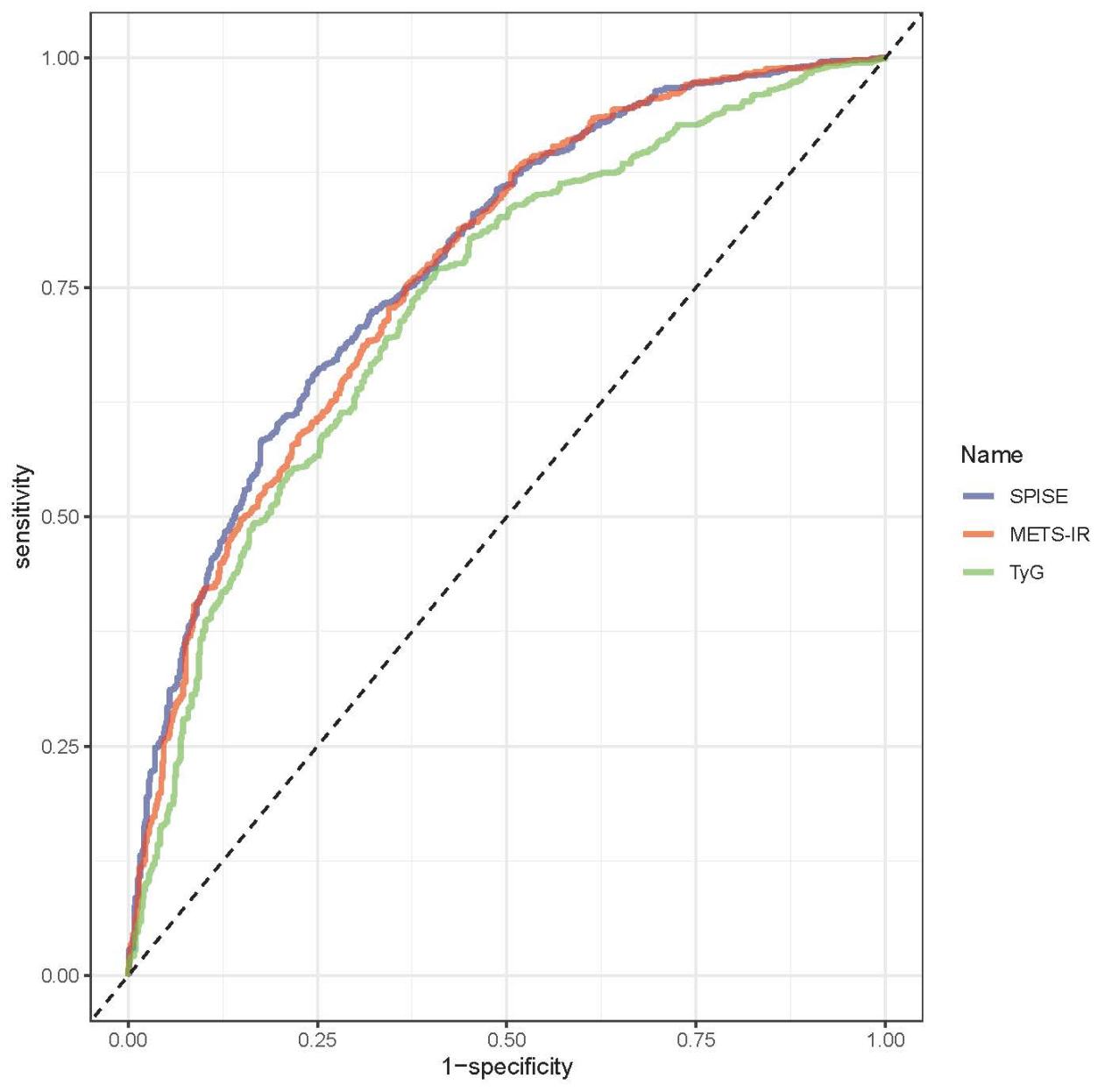
**

**Supplementary Table 3.​ Performance comparison of** **the SPISE index and two other insulin resistance indices (METS-IR and TyG index) for identifying NAFLD.**

| Variables | AUC (95% CI) | Accuracy (95% CI) | Sensitivity (95% CI) | Specificity (95% CI) | Cut off |
| --- | --- | --- | --- | --- | --- |
| SPISE index | 0.78 (0.75 – 0.80) | 0.69 (0.67 – 0.72) | 0.66 (0.63 – 0.69) | 0.75 (0.72 – 0.79) | 6.007 |
| METS-IR | 0.77 (0.74 – 0.79) | 0.71 (0.68 – 0.73) | 0.63 (0.59 – 0.67) | 0.75 (0.73 – 0.78) | 37.624 |
| TyG index | 0.73 (0.71 – 0.76) | 0.70 (0.68 – 0.72) | 0.59 (0.55 – 0.63) | 0.77 (0.74 – 0.80) | 8.472 |

**Supplementary Table 4.​ Pairwise comparison of the AUC between the SPISE index and two other insulin resistance indices (METS-IR and TyG index) for identifying NAFLD.**

| Categories | ∆ AUC (95% CI) | SD | *Z*-value | *P*-value |
| --- | --- | --- | --- | --- |
| SPISE index *vs.* METS-IR | 0.0105 (0.00327 – 0.0176) | 0.00367 | 2.850 | 0.0044 |
| SPISE index *vs.* TyG index | 0.0442 (0.0255 – 0.0629) | 0.00956 | 4.622 | < 0.0001 |
